# Degradation of Phosphate Ester Hydraulic Fluid in Power Station Turbines Investigated by a Three-Magnet Unilateral Magnet Array

**DOI:** 10.3390/s140406797

**Published:** 2014-04-14

**Authors:** Pan Guo, Wei He, Juan C. García-Naranjo

**Affiliations:** 1 State Key Laboratory of Power Transmission Equipment & System Security and New Technology, Chongqing University, Chongqing 400044, China; E-Mail: hewei@cqu.edu.cn; 2 MRI Centre, Department of Physics, P.O. Box 4400, University of New Brunswick, Fredericton, NB E3B 5A3, Canada; 3 Centre of Medical Biophysics, Universidad de Oriente, Patricio Lumumba S/N, Santiago de Cuba 90500, Cuba; E-Mail: juan.garcia@cbiomed.cu

**Keywords:** three-magnet array, unilateral magnetic resonance, turbine oils, power station, aging accessment, T_2eff,long_, T_1,long_, rejection standard

## Abstract

A three-magnet array unilateral NMR sensor with a homogeneous sensitive spot was employed for assessing aging of the turbine oils used in two different power stations. The Carr-Purcell-Meiboom-Gill (CPMG) sequence and Inversion Recovery-prepared CPMG were employed for measuring the ^1^H-NMR transverse and longitudinal relaxation times of turbine oils with different service status. Two signal components with different lifetimes were obtained by processing the transverse relaxation curves with a numeric program based on the Inverse Laplace Transformation. The long lifetime components of the transverse relaxation time T_2eff_ and longitudinal relaxation time T_1_ were chosen to monitor the hydraulic fluid aging. The results demonstrate that an increase of the service time of the turbine oils clearly results in a decrease of T_2eff,long_ and T_1,long_. This indicates that the T_2eff,long_ and T_1,long_ relaxation times, obtained from the unilateral magnetic resonance measurements, can be applied as indices for degradation of the hydraulic fluid in power station turbines.

## 1. Introduction

Since the discovery of their excellent anti-wear and fire resistance properties in the 1940s, the use of phosphate ester hydraulic fluids by industry has steadily increased [1,2]. In power systems, phosphate esters are used primarily as fire-resistant base-stocks in turbines for speed governing, lubricating, radiating, cleaning and vibration damping [3]. In operation at high temperatures, in the presence of oxygen, water vapor and catalytically active metals, these synthetic oils are severely stressed. These conditions may lead to a rapid degradation of their oxidation and the hydrolysis resistance, which may cause corrosion and even failure of the system [4–6]. In order to prevent change of viscosity, formation of deposits and corrosion, oil degradation must be minimized [7]. Therefore, the hydraulic system is usually equiped with a by-pass regenerating unit [3] to remove acidic substances and water, as well as solid particulate contaminants. During the aging process, the oil continuously degrades and is regenerated until it is non-renewable by accumulation of degradation products. It is very expensive (about 110,000 USD) to replace the oil in power plant turbines [8]. Consequently, a quality control measurement is needed for safe and economic operation of the power system.

With deterioration [8] of phosphate ester fire resistant oils the color deepens and precipitates are produced in severe cases. During aging, the acidity, water content, viscosity, dielectric constant, foam characteristic, and air release property, *etc.* may all change [7,9].

Traditionally phosphate ester aging was evaluated by observing color and turbidity changes [3]. This method is less rigorous, and sometimes led to major accidents and resulted in irreparable damage and significant economic losses. More recently turbine operators are required to test the appearance, acidity, resistivity and mechanical impurities of the oil at least once a week, and measure the moisture content, flash point and viscosity at least once per season [9]. Some sites even test the thermo-oxidative stability and corrosivity under simulated aging conditions, where the increase of acidity and viscosity, the formation of sludge and corrosion against various metals are controlled [7]. However, such a test program is expensive and time consuming and cannot be applied to a large number of samples in the surveillance of used oils.

NMR has been used in the past to investigate materials aging and degradation. A prominent and most practical example is the development of unilateral magnetic resonance (UMR), where the NMR experiment is carried out in the inhomogeneous field produced on one side of a portable magnet. UMR has become a powerful technique in different areas of application. New applications [10] have been developed in well-logging [11], biomedicine [12], material analysis [13] and characterization of food products [14]. Since UMR is simpler and much less expensive than traditional NMR, and produces reliable information, the development of new UMR sensors and applications should continue in the near future.

This paper presents a magnetic resonance method for phosphate ester analysis employing a three-magnet array [15,16] as a sensor. The intention of this work is to develop a rapid and simple method for estimating the degree of degradation of phosphate ester hydraulic fluids in power plant turbines. We have demonstrated that the new sensor produces reliable results and can be employed to follow the oil aging process. In the following sections, the features of the magnet and measurements on phosphate ester fire resistant oils are discussed.

## 2. Experimental Section

### 2.1. Magnet

Magnets of different types can be employed for this measurement. Closed or semi-closed magnets allow more sensitive measurements than unilateral magnets, but require of a more complicated design and adjusting process and are more sensitive to temperature variation. Unilateral magnets are in general very simple to build and can also produce reliable results.

**Figure 1.**
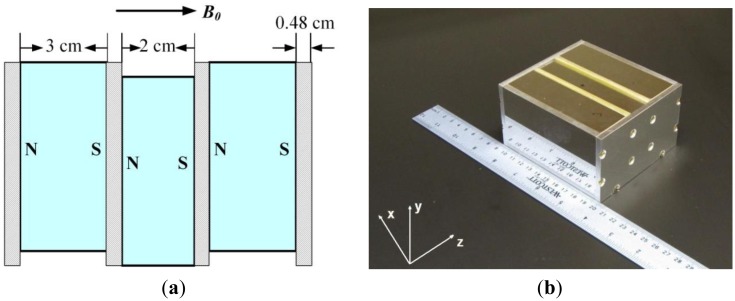
Schematic (**a**) and photo (**b**) of the three-magnet array. The centre of the upper surface of the magnet array corresponds to the position (0,0,0) in the coordinate system.

Figure 1 shows the structure of the three-magnet array unilateral magnet developed by the UNB MRI Centre in Canada. It is a simple array of three magnet blocks with the magnetic field oriented in the same direction. A vertical displacement of the central block allows generating a homogeneous spot [15] or an extended constant gradient [16]. The design is compact and safe and the weight of the magnet array is 5 kg. The mathematical equations to describe the magnetic field distribution are relatively simple, which makes easy the simulation. It should be marked that the magnetic field homogeneity (around 1% of B_0_) for the homogeneous spot design is still far away from the values employed for classic NMR experiments. The major advantages of this magnet are its simplity and the relatively remote homogenenous spot. The static magnetic field B_0_ is parallel to its surface (along z axis in Figure 1) which allows employment of a very simple surface coil with good sensitivity. The size of the measurement spot results from the combination of B_0_ distribution, bandwidth of the excitation RF pulses, bandwidth of the receiver and parameters of the surface coil like size, shape and quality factor (Q).

**Figure 2.**
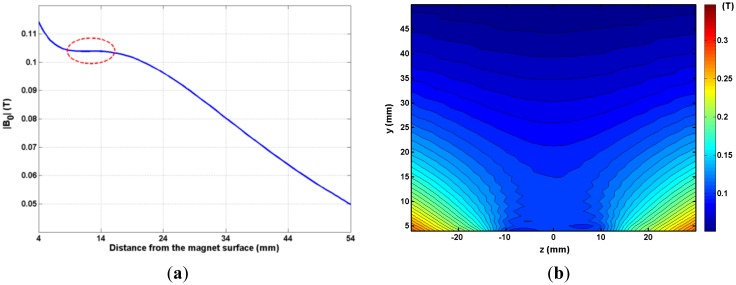
(**a**) Plot of magnetic field magnitude B_0_ as a function of the distance from the centre of the magnet surface. The circled area indicates the sensitive spot position; (**b**) Contour plot of the magnetic field magnitude B_0_ in the yz plane. The field is reasonably symmetric.

In order to adjust and characterize the magnet, magnetic field measurements were undertaken employing a three axis Hall effect magnetic field probe (Lake-Shore Cryotronics Inc., OH, USA) and a computer controlled three axis plotter (Velmex Inc., MI, USA). Figure 2a plots the magnetic field magnitude as a function of distance from the centre of the magnet. The sensitive spot of the magnet array is 8 mm to 17 mm from the magnet surface. The proton resonance frequency at this position is 4.485 MHz. Figure 2b shows a contour plot of the magnetic field along the yz plane over the magnet (x = 0).

### 2.2. RF Coil

**Figure 3.**
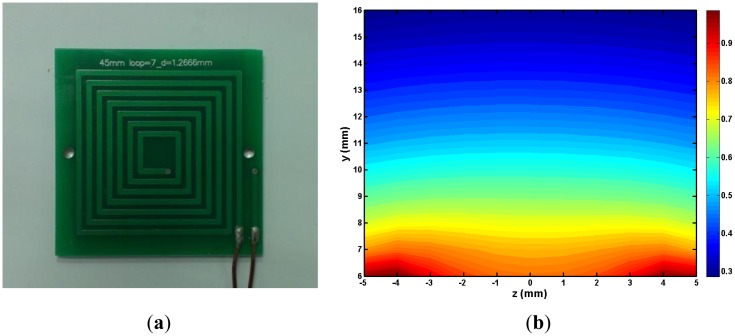
(**a**) Photo of the RF coil; (**b**) The simulated result of the normalized RF field distribution in the central perpendicular plane. The B_1_ field is perpendicular to the coil. y = 6 mm is the upper surface of the RF coil.

A square spiral RF coil, 45 mm in length with 7 turns, fabricated on a 1.2 mm thick printed circuit board (Figure 3a) was employed for the measurements since the RF field B_1_ is required to be perpendicular to the static magnetic field B_0_.The lead width was 1.5 mm and the spacing 1.27 mm. The resistance and inductance of the coil were 0.41 Ω and 1.439 μH, respectively. The loaded quality factor (Q_L_), measured with the coil placed on the magnet was 30. The RF coil was tuned 4.485 MHz, which is the proton resonance frequency at the centre of the spot. The dead time of the coil is 35 us. The RF field above the coil, simulated employing the simulation software Maxwell 3D (Ansoft, Pittsburgh, PA, USA), is shown in Figure 3b. A 4.766 mm (3/16″) fiberglass spacer was placed between the coil and the magnet to assure a better use of the homogeneous spot of the magnet and the B_1_ of the coil. The distance from the RF coil upper surface to the sensitive spot is 2 mm to 11 mm.

### 2.3. Experiment Details

Two groups of turbine phosphate ester hydraulic fluids (Table 1), in service at two different power stations, were employed for the measurements. They were housed in five cylindrical glass vessels. The dimensions (4 cm in diameter and 7 cm in length) of the glass vessels were chosen to guarantee full coverage of the measurement spot (1 cm along z axis, 1 cm along y axis and 2 cm along x axis) with the sample.

All measurements were carried out with a Kea2 console (Magritek, Wellington, New Zealand), connected to a RF power amplifier (TOMCO Technologies, Stepney, Australia) at 17 ± 0.3 °C. The ^1^H NMR transverse relaxation time (T_2eff_) [17] and longitudinal relaxation time (T_1_) were obtained with the standard Carr-Purcell-Meiboom-Gill (CPMG) sequence and T_1_ Inversion Recovery with CPMG T_1_ IR Add sequence, respectively. For the CPMG sequence, the 90° pulse width was 8 μs, the attenuations of the 90° and 180° pulse were −18 dB and −12 dB, the echo time was 150 μs, the number of echoes was 2,048, the repetition time was 400 ms, and 256 scans were averaged. The total measurement time was 5 min. For the T_1_ IR Add sequence, the time between the first 180° pulse and the 90° pulse was varied from 1 ms to 400 ms by log spacing with 40 steps, the number of echoes was 32, and other parameters were the same as in the CPMG sequence. The total measurement time was 3 h.

## 3. Results and Discussion

### 3.1. Transverse Relaxation Time Measurements

**Figure 4.**
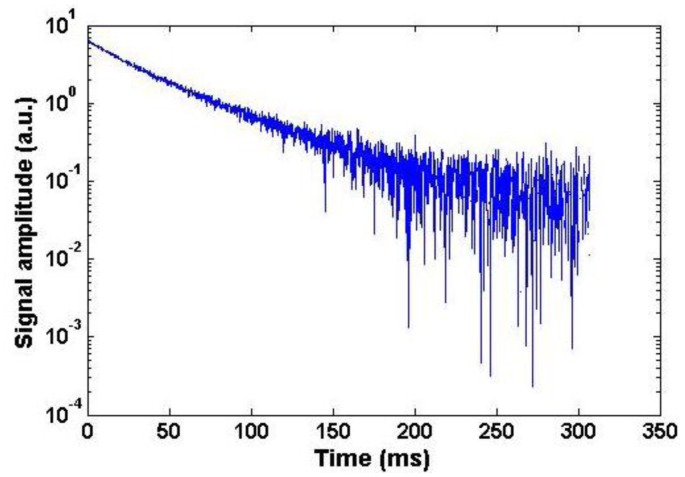
Semi-logarithmic plot of a CMPG decay from a sample of turbine hydraulic fluid.

**Figure 5.**
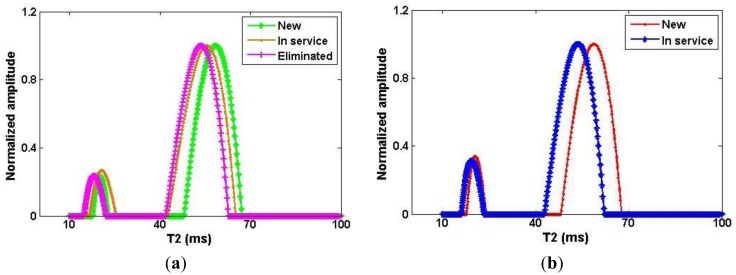
T_2eff_ distributions from the CPMG measurement for the Beilun power station (**a**) and the Yuyao power station (**b**).

Once the CPMG decay from the sample (Figure 4) was obtained, an Inverse Laplace Transformation (ILT) of the CPMG data was performed with the Contin program [18]. In Figure 5 the T_2eff_ distributions of two groups of turbine oils are shown. The amplitude of the short lifetime component is approximately three times less than the long component, so that the CPMG decay is dominated by the longer transverse relaxation time, T_2eff,long_. The measurement was repeated five times for each sample to check the reproducibility of the method. As the reproducibility of the long component is much better than the short component, only T_2eff,long_ will be discussed hereafter.

In Figure 5, all of the T_2eff_ distribution curves have two symmetric peaks that are well separated. This behavior allows a much simpler bi-exponential fitting to be employed. In addition to more reliable results the simplicity of the bi-exponential fitting (Equation (1)) [19] makes it better option for practical measurements. Only the T_2eff,long_ obtained from the bi-exponential fit was chosen for display to show the contribution of the more representative component inside the oil (Table 2). The aging status of different turbine oils can be distinguished in the measured T_2eff,long_ and an increase in service time yields a decrease in transverse relaxation time T_2eff,long_. The differences in T_2eff,long_ are not large but they are reproducible and reliable:

(1) $$M(t)=A_{\text{short}}\exp\left(-\frac{t}{T_{2\text{eff},\text{short}}}\right)+A_{\text{long}}\exp\left(-\frac{t}{T_{2\text{eff},\text{long}}}\right)$$

### 3.2. Longitudinal Relaxation Time Measurements

Since in an inhomogeneous magnetic environment it is practically impossible to obtain FID signals, inversion recovery as described by Hurliman in [20] with CPMG added, as implemented in the Prospa software (Magritek, Wellington, New Zealand) was employed as the sequence for measuring T_1_ of the oils. The CPMG echo train was summed on the spectrometer before being returned to the software Prospa. The integrals of the CPMG echoes were fitted to Equation (2):

(2) $$A=A_{0}\left(a*\exp\left(-\frac{\text{t}}{{\text{T}}_{1,\text{short}}}\right)+b*\exp\left(-\frac{t}{T_{1,\text{long}}}\right)\right)$$

**Figure 6.**
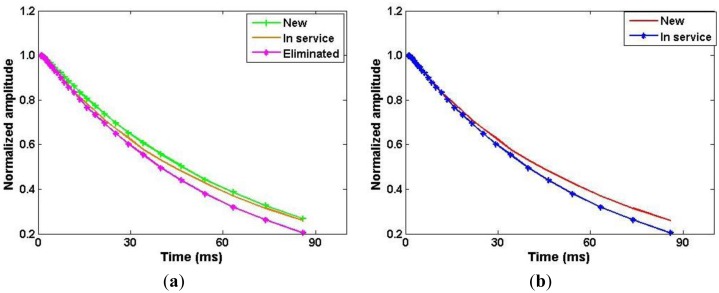
T_1_ decay curves of the turbine oils of the Beilun power station (**a**) and the Yuyao power station (**b**).

For clarity, only the first 30 points of the curves are shown in Figure 6. As the reproducibility of the short component was not reliable, only the long component T_1,long_ was chosen to represent the longitudinal relaxation time of the turbine oils. From Table 3, an increase in the service time yields a decrease in longitudinal relaxation time.

## 4. Conclusions

The aging status of phosphate ester hydraulic fluids with different service times, from two different power stations, has been studied by ^1^H relaxation time measurements with a three-magnet array unilateral magnet as a sensor. We demonstrate that the aging results in a decrease in T_2eff,long_ and T_1,long_ relaxation times. Therefore, T_2eff,long_ and T_1,long_ can be used as the indices of turbine oil aging. This method is simple and produces reliable results. The next step will focus on the measurements of more turbine oils to establish a statistical data base and measurements of other sample parameters, such as molecular diffusion, with unilateral magnetic resonance. We propose this method to predict when the turbine oils should be eliminated to prevent unexpected accidents in power stations.

## Figures and Tables

**Table 1.** Turbine hydraulic fluid for the measurements.

**Power Station Name**	**State**
Beilun	New
	In service
	Eliminated
Yuyao	New
	In service

**Table 2.** The T_2eff,long_ of different turbine oils extracted from bi-exponential fitting.

**Power Station Name**	**Status**	**T_2eff,long_ (ms)**
Beilun	New	75.8 ± 0.9
	In service	65.8 ± 0.5
	Eliminated	61.9 ± 0.8
Yuyao	New	66.9 ± 0.7
	In service	60.8 ± 1.1

**Table 3.** The T_1,long_ of different turbine oils extracted from the T_1_ distribution curves.

**Power Station Name**	**Status**	**T_1,long_ (ms)**
Beilun	New	73 ± 1
	In service	69.5 ± 0.7
	Eliminated	64.5 ± 0.6
Yuyao	New	62 ± 0.6
	In service	53 ± 2.0
